# Midwives' Experiences of Providing the “Inspirational Lecture” as a Care Intervention for Expectant Parents—A Qualitative Study

**DOI:** 10.3389/fpubh.2020.575062

**Published:** 2020-10-22

**Authors:** Caroline Bäckström, Tina Söderlund, Stina Thorstensson, Lena B. Mårtensson, Marie Golsäter

**Affiliations:** ^1^School of Health Sciences, University of Skövde, Skövde, Sweden; ^2^Skaraborg Hospital Skövde, “Woman, Child” (K3), Skövde, Sweden; ^3^Futurum, Academy for Health and Care, Jönköping, Sweden; ^4^CHILD-Research Group, School of Health and Welfare, Jönköping University, Jönköping, Sweden

**Keywords:** transition, pregnancy, childbirth experience, parenthood, content analysis, antenatal, parental education

## Abstract

**Background:** In most Western countries, ordinary parental classes exist and have become a well-established form of professional support within midwifery care, even though some of these classes lack evidence of benefits for the parents. A Swedish randomized controlled trial including an intervention as a pilot study, revealed that a type of parental preparatory professional support provided for expectant parents, the “inspirational lecture,” showed a tendency to be beneficial for parents' birth experience, and their perceived quality of parental couple relationship. However, there is no previous research on the midwives' experiences from providing the inspirational lecture. Carrying out research on midwives' experiences from providing the lecture, could bring future opportunities to provide a work-integrated learning (WIL) related to professionals' skills, and the pedagogic used.

**Aim:** To elucidate midwives' experiences about providing the inspirational lecture as a care intervention for expectant parents.

**Methods:** Midwives were interviewed and data were analyzed using qualitative content analysis.

**Results:** The midwives strived to put childbirth into a comprehensive and manageable context for the expectant parents, during the inspirational lecture. For this, different approaches were used to make expectant parents understand how the parents themselves can be engaged participants in their own birth.

**Conclusion and Clinical Implications:** The midwives used the inspirational lecture to provide the expectant parents with knowledge about how they, as a parental couple, could cooperate and feel safe in relation to the upcoming birth. This could be understood as if the midwives were striving to facilitate the integrative power of the parental couple, which is the couples' ability to gather their joint power. These results can assist midwives and serve as a reference for providing parental classes for expectant parents with a focus on promoting both the parents' individual as well as mutual skills.

## Introduction

In most Western countries, ordinary parental classes exist and have become a well-established form of professional support within midwifery care (1), even though evidence around the benefits of these classes for parents is lacking (1, 2). Furthermore, expectant parents lack satisfactory professional support in relation to their needs of preparation for childbirth and parenthood (3, 4). On the contrary, attendance at childbirth preparation has been associated with more women experiencing spontaneous labor onset and arriving at the hospital in active labor (5).

The extent to which ordinary parental classes are available for expectant parents, however, varies both internationally and nationally. Nationally, in Sweden, expectant parents are offered ordinary parental classes to a varying degree even though a national commission has recommended these classes for all expectant parents since 1979 (6). Currently, both small and large-group variations of ordinary parental classes are offered to expectant parents within Sweden (7). Most commonly, midwives are the leaders of these ordinary parental classes and provide expectant parents with information on how to prepare for childbirth, breastfeeding, and parenthood. A Swedish randomized controlled trial including an intervention as a pilot study allocated expecting parents to receive: (1) parental preparatory professional support provided by midwives in large groups of parents, the “inspirational lecture,” in combination with ordinary parental classes provided by midwives in small groups of parents (intervention group, *n* = 66), or (2) ordinary parental classes provided by midwives in small groups of parents (control group, *n* = 60). The results of the study revealed that the intervention, the inspirational lecture, showed a tendency to be beneficial for parents' birth experience, and there was a statistically significant difference showing that the parents within the intervention group reported higher values regarding their perceived quality of parental couple relationship, as well as their manageability. The authors' conclusion was that a combination between those two types of professional support (the inspirational lecture and ordinary parental classes), seems to be a valuable care intervention (8). Qualitative research on expectant first-time mothers and partners' perceptions of the inspirational lecture have shown that the pedagogical creativity used by the midwives who provide the lecture (i.e., conveying information using humorous role-playing), increase understanding, among the expectant parents (3, 4). However, there is no previous research on the midwives' experiences from providing the inspirational lecture.

Previous research has shown that the leadership role of parental classes seems important in terms of how parents experience the group sessions and if their parental role is affected by them after birth (9). Also, midwives' experiences from leading ordinary parental classes have been described both as demanding (10) and enjoyable (11), and midwives need both individual skills and organizational resources to provide satisfactory parental classes (10, 11). When developing the inspirational lecture, provided as an intervention within the Swedish RCT previously mentioned (8), the intent was to create opportunities for midwives to provide expectant parents with information about how to prepare for normal childbirth. The developers used their clinical experiences from working as labor ward midwives. In addition, the inspirational lecture was developed to provide expectant parents with information about how they can prepare for childbirth, with the focus on understanding how to manage: different labor phases (i.e., latent phase and active phase) and non-pharmacological pain relief (breathing and massage techniques, etc.). The inspirational lecture is provided for a large group of parents, approximately 80–120 parents attending, for 2 h. Further, it is an open lecture, which means that the parents do not have to apply for it and they can attend as many times as they need. Expectant parents as a couple or pregnant women together with a friend, sister, or significant other are all welcomed. In this study, the term *expectant parents* will be used when referring to attendees to the inspirational lecture, even though it is a somewhat narrow description. As mentioned, there is no previous research on midwives' experiences from providing the lecture, and such research could generate knowledge of how midwives use their professional skills when developing, introducing and providing professional support as care interventions for expectant parents.

Carrying out research on midwives' experiences from providing the inspirational lecture, could bring future opportunities to provide a work-integrated learning (WIL) related to professionals' skills, and the pedagogic used by the midwives as well (12). According to Lagrosen et al. (13), a WIL approach stimulate learning through the active exchange of knowledge and reflected action. Furthermore, Pennbrant and Svensson (12) suggest that the aim with WIL is to develop tools and knowledge that increase understanding about the conditions and processes related to educations and/or workplace learning, for example. Future research on midwives' experiences from providing the inspirational lecture could be seen as an interdisciplinary combination between the expertise of the midwives' clinical experiences and skills, and research-based clinical evidence that could increase opportunities to achieve future high-quality care (12). Also, knowledge about midwives' experiences of providing this type of parental class could contribute valuable knowledge for midwives who provide parental classes internationally. This is because, globally, as midwives provide professional support, they could gain further knowledge about how such care can promote health among the recipients. Therefore, the aim of the present study was to elucidate midwives' experiences about providing the inspirational lecture as a care intervention for expectant parents.

## Materials and Methods

An explorative qualitative design using interviews (14) and a qualitative content analysis (15) was chosen to elucidate midwives' experiences about providing the inspirational lecture as a parental class for expectant parents.

### Settings and Participants

The present study was undertaken in two counties in the southwest of Sweden with two hospitals that together have approximately 6,150 births annually. Both included hospitals separately provided the inspirational lecture for expectant parents. At the time of the present study, it was only the two included hospitals that provided the inspirational lecture for expectant parents. Altogether, eight midwives worked on providing the lecture. They provided the lecture in pairs, two pairs at each hospital. The Clinical Heads of Service in the childbirth wards approved access in order to undertake the study. These Clinical Heads of Service contacted the eight midwives to ask them about their interest in participating in the present study. All the midwives agreed to participate. The included midwives varied in clinical working experience between 5 and 25 years; their present workplaces were labor ward, antenatal ward, or a combination.

### Data Collection

Interviews were used to elucidate midwives' experiences about providing the inspirational lecture as a parental class for expectant parents. Prior to the interviews, all participants were contacted by the interviewer to inform about the study and the professional experience of the interviewer, as well as to establish a relationship between the participant and the interviewer. The interviewer had no previous relationship with the participants. The interviews with the midwives were conducted via telephone. All of the interviews followed a semi-structured interview guide with open-ended questions. These questions were used to encourage the midwives to describe their experiences and understanding. The open-ended questions were: *Could you describe the inspirational lecture? What is the purpose of the lecture? What is your role as a lecturer?* and *How do you experience the lecture?* When necessary, the interviewer asked follow-up questions for richer responses (14). Follow-up questions were, for example: *Could you explain more?* After each open-ended question, the interviewer made a brief summary that the midwives could comment on. This was done to let the midwives clarify the answers and the interviewer to confirm her interpretation (16). All interviews were held by one of the authors, between December 2015 and May 2016. The interviews were audio taped and lasted between 24 and 51 min. Two of the authors transcribed the interviews verbatim, 113 pages (A4) in all. All of the authors had a consensus that the interviews were rigorous and met the aim of the study.

### Data Analysis

Qualitative content analysis (15) was used for the data analysis process. First, each interview was read several times independently to allow each author to capture the content and essential structures of the interviews. Texts related to the aim of the study were highlighted, and open coding was conducted, which means that notes and headings were written in the text while reading it. After that, the headings were collected on coding sheets, and from these, codes were developed to explain the meaning of the text. Then, the codes were grouped into sub-categories based on similarities and differences of content. Three generic categories were developed, based on their underlying meanings. In the final step of the analysis, the main category arose (15). Three of the authors participated in each step of the analysis process. All five authors, with differing experiences of qualitative research, participated in discussions concerning the analysis, and agreed on a consensus once the analysis was complete. All of the authors participated in the process of writing the text. An overview of the analysis process is presented in Table 1.

**Table 1:** Overview with examples of the analysis process.

**Statements**	**Codes**	**Sub-categories**	**Generic categories**	**Main category**
*We present information seriously, mixed with humor, and we have noticed that humor is a very good way to reach out to the expectant parents*.	Present information seriously mixed with humor	Balancing humor and seriousness	Making childbirth understandable	Put childbirth into a comprehensible and manageable context
*The idea with the IL is that we should welcome them* [the expectant parents] *with open arms. And that they are free to come to the lecture one, two, or three times, if they want to. So I really like the concept*.	Make room for every pregnant woman and partner, and make each one of them feel welcomed	Create a sense of being welcomed	Promoting confidence in the childbirth process	
*They should not go to the delivery ward with the intention of *having us deliver their baby*. They should go to the delivery ward *to give birth*. They should think that we are there to strengthen them in their parenting and as a team. They should feel that they can manage it as a team, and that we are there to support them when they are giving birth*.	The midwives strive to strengthen the pregnant women and their partners as a team	Promote cooperation among the expectant parental couple	Facilitating the expectant parental couple's cooperation during childbirth	

### Ethical Considerations

The interviews were performed according to the Swedish law stating that ethical approval is not needed when interviewing health care professionals about work-related questions (17). The Clinical Heads of Service in the childbirth wards approved access to undertake the study and provided the researchers with contact information for midwives that met the inclusion criteria. After that, the researchers contacted the midwives to inform about the study and ask for their willingness to participate. When designing the study, the potential risks were deliberated. The participants were not considered to represent a vulnerable group of individuals; their health was not considered negatively risked by participating in interviews. Thus, the potential benefits of the study were considered to outweigh any risks. To protect participant's safety and rights, though, the Declaration of Helsinki (18) was followed, which allowed the participants: to receive information about the study and how data would be handled; right to self-determination, informed consent, and ability to withdraw participation at any time, as well as; confidentiality of personal information. The participants gave their consent to the publication of their quotes. Within the results section, quotes are provided in italics with an individual code (M1–M8) for each individual participant.

## Results

The analysis resulted in the following main category: *Put childbirth into a comprehensive and manageable context*. Three generic categories were found: *Making childbirth understandable; Promoting confidence in the childbirth process;* and *Facilitating the expectant parental couple's cooperation during childbirth*. Each generic category is based on two sub-categories, as presented in Table 2.

**Table 2:** Overview of main category, generic categories, and sub-categories.

**Main category**	**Generic categories**	**Sub-categories**
Put childbirth into a comprehensible and manageable context	Making childbirth understandable	Use practical illustrations
		Balancing humor and seriousness
	Promoting confidence in the childbirth process	Create feelings of comfort and security
		Create a sense of being welcomed
	Facilitating the expectant parental couple's cooperation during childbirth	Promote cooperation between the expectant parental couple
		Create partner involvement

### Main Category: Put Childbirth Into a Comprehensible and Manageable Context

According to the midwives', the intent of providing the inspirational lecture as a parental class for expectant parents was to put childbirth into a comprehensive and manageable context for the expectant parents. To achieve this, the midwives used different approaches to explain childbirth and the valuable roles played by the pregnant women and their partners. The midwives thought that if the parents understood how to prepare for childbirth, and if they understood their individual roles in the “birthing team,” it would help them to manage the upcoming childbirth. The midwives thought that when expectant parents understood the childbirth process and felt competent enough to manage it, this would increase the chance for positive childbirth experiences. Consequently, the midwives also used different approaches to facilitate cooperation in the expectant parental couple. Altogether, the midwives presented the complexity of childbirth in an easygoing and positive way, to make expectant parents understand, feel positive about, and confident in the childbirth process.

#### Making Childbirth Understandable

According to the midwives, expectant parents need to understand childbirth in order to be prepared for it. Therefore, the midwives used practical illustrations, which included a balance between humor and seriousness to promote understanding about how to prepare for childbirth.

##### Use practical illustrations

To promote expectant parents' understanding of what situations they might be involved in when giving birth, the midwives used practical illustrations to clarify the process. For example, they showed how the expectant parents could use different practical things such as massage tools or a Pilates ball that might be used during childbirth. The midwives also wore the same clothing during the inspirational lecture as the midwives in the labor ward do. They did this with the intention to make the expectant parents feel safe from recognizing the clothing, when arriving in the labor ward to give birth.

Furthermore, the midwives role-played to illustrate in practice different situations that might occur during childbirth. During these role-plays, one of the two midwives who provided the inspirational lecture act as a birthing woman while the other one act as a partner. Then, the midwives were able to show different outcomes of the same situation, where the outcome depended on how the pregnant women and their partners acted in the exemplified situation (midwives acting to illustrate stressed or anxious expectant parents vs. midwives acting to illustrate calm or confident expectant parents).

To make childbirth understandable, the midwives not only explained how the expectant parents could act but also why. The midwives emphasized that knowing *why* to act was valuable because when expectant parents understood the underlying reason for acting, it promoted their understanding. These situations could be about the importance of nutrition, elimination, physical activity, and resting during childbirth, both from the perspective of the birthing woman and her partner.

…*the word why is essential for us* [the midwives who provide the Inspirational lecture]. *For example, we say that it is important, what should I say… to pee… WHY is it important that you pee? Not just that, this is how it is… so that they will understand. They* [the parents] *are more motivated when they know what will happen if they do not do that* [pee] (M 6).

The midwives experienced that different types of practical illustrations, combined with oral explanations, could facilitate understanding among the expectant parents. The visualization these illustrations provided was to facilitate for the expectant parents to remember and relate to the given information. Through this, the midwives described their intention to make the complexity of childbirth understandable for expectant parents, as well as help them understand how they could act to manage childbirth.

*... we tell the expectant parents that their baby is supposed to rotate in the birth canal, and therefore it is good that the woman varies her postures during childbirth. We demonstrate this by using our hands to show what it looks like when you take off a* [stiff] *bracelet. You cannot just pull your arm and hand right out of it. You have to rotate your arm and hand to remove the bracelet*. (M 1).

##### Balancing humor and seriousness

According to the midwives', using humor when presenting information could make the expectant parents laugh at something they were actually nervous about (childbirth). In the following, this could help the expectant parents relax and be more open to information during the inspirational lecture: “*We present information seriously, mixed with humor, and we noticed that humor is a very good way to reach out to the expectant parents”* (M 7).

When using humor, the midwives tried to create a relaxed atmosphere during the inspirational lecture. The underlying idea was that, if the expectant couples were able to relax during the inspirational lecture, it would be helpful for them to be able to relax also in their preparation for childbirth. The midwives thought that relaxation, as well as laughing, helped the expectant parents to handle or to release childbirth fears. Furthermore, relaxation and laughing were assumed by the midwives to facilitate understanding and learning abilities. However, the midwives described how it was important for them to balance humor with seriousness, to maintain the professionalism of the inspirational lecture.

#### Promoting Confidence in the Childbirth Process

During the inspirational lecture, the midwives tried to promote confidence in the childbirth process. To do this, the midwives experienced that it was important for them to create feelings of comfort and security as well as to create a sense of feeling welcomed in the expectant parents.

##### Create feelings of comfort and security

The midwives described how they tried to create feelings of comfort and security. They wanted the expectant parents to perceive the midwives as trustworthy, because they saw themselves as representatives of other midwife colleagues. To achieve this, they expressed the value of being interested in inspiring expectant parents for childbirth. Furthermore, as presenters they needed to take an interest in performing as well as liking to perform the specific concept of the inspirational lecture *and* to represent the midwifery profession.

*I work as a midwife in the labor ward... We* [the midwives providing the Inspirational Lecture] *are representatives of all our colleagues. I want them* [the expectant parents] *to feel like “Oh, I want to meet them* [midwives] *in the labor ward. I look forward to meeting them* [midwives] *because they* [midwives] *think it* [childbirth] *is fun and I will feel like that as well, when it is my turn to give birth.”* (M 6)

During the inspirational lecture, the midwives also proposed to the expectant parents that they should create an individual mental goal. This mental goal could then be used by the parents to cope with childbirth. The midwives thought that a mental goal could work as a positive reminder of why the parents wanted to experience childbirth; it could work as a positive reminder of the baby. Altogether, this was supposed to help the expectant parents in their feelings of excitement and mental preparation for childbirth. For this, the inspirational lecture was presented in an easygoing and positive way to make expectant parents feel confident in the childbirth process.

*So, the inspiration itself is, probably, about us weaving in a little of reality; how it can be…* [we] *provide them with mental goal targets so that they can make an image in their head, which they can feel almost inspired from* [giving birth] (M 5).

##### Create a sense of being welcomed

The midwives described the importance of creating an atmosphere where the expectant parents felt that they were welcomed to the inspirational lecture. The midwives saw and welcomed each one of them who arrived at the inspirational lecture, in person. The midwives thought that the expectant parents' feelings of being welcomed to the lecture could affect their feelings of being welcomed to the labor ward to give birth as well.

*I want to convey such a positive feeling, so that they* [the expectant parents] *feel welcomed. They should feel reassured, seen, and so on. For example, we stand in the entrance when they* [the expectant parents] *arrive and say things like “Hello, welcome!”*... (M 6)

To further create a sense of being welcomed, the inspirational lectures were held in large lecture halls, to enable everyone who came to attend the lecture. This was described by the midwives as a way of promoting feelings of having room for everyone, both in the lecture hall and in the labor ward. Further, the inspirational lecture was free of charge, scheduled in the evenings to match the expectant parents' working hours, and they could attend the inspirational lecture as many times as they wished.

#### Facilitating the Expectant Parental Couple's Cooperation During Childbirth

The midwives explained that one important goal of the lecture was to facilitate the expectant parental couple's cooperation during the childbirth process. To achieve this, a major part of the inspirational lecture focused on the partner, to create partner involvement and to promote the cooperation among the expectant parental couple.

##### Promote cooperation between the expectant parental couple

The midwives described their intention to promote cooperation between the expectant parental couple. This cooperation could create feelings of internal power within the parental couple, according to the midwives' experiences. This internal power was about the pregnant woman and her partner feeling able to manage birth together and was exemplified as: “We can do it together as a team” or “What an amazing thing we have in front of us.”

*They* [the expectant parents] *should not go to the labor ward with the intention of having us* [midwives] *deliver their baby*. *They should go to the labor ward to give birth*. *They should think that we* [midwives] *are there to strengthen them in their mutual parenting. They should feel that they* [the expectant parental couple] *can manage it* [childbirth and parenting] *as a team, and that we are there to support them when they are giving birth*. (M 1)

To create such feelings of internal power, the midwives talked about childbirth with excitement. The midwives assumed that when they presented positive feelings during the inspirational lecture, it could strengthen the expectant parents and increase their feelings of excitement about childbirth as well as their feelings of being able to manage childbirth together. Furthermore, as the inspirational lecture was given as a large group parental class, many expectant parents gathered together in the same area. This could help the parents to feel that they were not alone in having childbirth ahead of them, according to the midwives'. This was thought to facilitate feelings of being able to manage childbirth in the expectant parents.

*It's the mentality that the parents feel secure and they believe in their own ability to give birth* [M 2]

##### Create partner involvement

The midwives described that, during the inspirational lecture, the partners were as important as the pregnant/birthing women. The midwives' intention was to confirm the partner's valuable role in the “birthing team.” Through this, the partner and the birthing woman could work together to cope with and experience childbirth. For this to work, the partners had to get involved and take an active role, according to the midwives'. To achieve this, the midwives explained to the partner how to be committed to this and play an active role. This was, for example, done by providing information about how to support women during childbirth, and how the partners could cope with different childbirth situations.

*We focus a lot on the partner... in this Inspirational Lecture... Partners have come to us afterwards and said that “Oh, this is exactly what I have been missing”; “You cannot read this on the Internet”; and “I thought that I couldn't do anything... that I should just sit beside, on a chair... that I should be. totally helpless and not able to do anything.” And they* [the partners] *have expressed “thank you” and “now we know what to do.”* (M 2)

The midwives experienced it as essential to encourage the partners to see themselves as important. To achieve this, they reassured the partners that professionals in the labor ward valued the partners and were available to support them. To make each partner visible, the midwives used the term “partners” instead of “fathers.” This was because a partner could, according to the midwives, be anyone: the expectant father or co-mother, a relative or friend, for example.

*Because that's what it's all about, whether it's a father or a family member in a family relationship, or if it is a partner who is of the same sex, all of them are supposed to get the same attention; it's their experience as well*. (M 5)

In summary, the midwives saw the partner as the person/s that the pregnant woman had chosen to support her during childbirth. The midwives' intention with this was to treat all partners equally, regardless of their gender or relationship to the pregnant woman.

## Discussion

In the present study, midwives' experiences about providing the inspirational lecture as a parental class for expectant parents were elucidated. The main findings were that the midwives wanted to put childbirth into a comprehensive and manageable context for expectant parents. To achieve this, the midwives used their theoretical knowledge and clinical experiences as a base for the inspirational lecture. This is similar to WIL which has been described earlier (12). Further, the midwives explained that they encouraged the expectant parental couple to see themselves as a “birthing team,” with valuable individual roles and an internal power focused to reach the same “mental goal.” Also, the midwives experienced it as vital to make the partner's role visible. It is a well-established fact that both women and partners want professional support to include the partner's role and focus on the expectant parents as a couple (3, 4, 7, 19–21). Current findings highlight that the inspirational lecture as a parental class can facilitate the expectant parental couple's cooperation during childbirth. For this, the midwives role-played to practically illustrate different outcomes of the same situation. The outcome depended on how the expectant mothers and their partners acted in the exemplified situation (stressed or anxious expectant parents vs. calm or confident expectant parents). This brings valuable knowledge into the field, since it shows how midwives strive to make expectant parents understand how the parents themselves can be engaged participants in their own birth. It could be understood as facilitating integrative power that is the couples' ability to gather their joint power (22). To be able to become engaged participants, however, the parents' abilities to comprehend childbirth might be vital for their abilities to manage the same. Nevertheless, to gain more knowledge about how midwives can support parents in becoming engaged participants in their own birth, further exploration is needed.

The midwives within the present study were aware that they (the midwives) themselves had to provide information with security and confidence to facilitate feelings of security among the pregnant women and their partners. This points to the importance of presenters being secure in their professional knowledge (23, 24). Previous research shows, on the other hand, that midwives (10) and other health professionals (9) find leading parental classes to be challenging. Also, midwives have stated that they lack the knowledge and resources to lead parental classes (10, 11). The current findings contribute to further knowledge about how midwives approach their task of leading (or teaching) such parental classes. The approach used by the midwives who provided the inspirational lecture could be described as focused on visualizing, through practical illustrations, how the parents could become engaged participants in their own birth. Besides this, the approach was based on the midwives' use of humor. The midwives experienced that the pregnant women's and partners' relaxation during the inspirational lecture was facilitated when information was provided through practical illustrations or humor. Moreover, this relaxation contributed to the parents' comprehension, according to the midwives. Humor has earlier been explained to create feelings of security in stressful childbirth situations (25). In addition, humor and role-play can improve learning as well, because they facilitate attention and interest in understanding information (26).

The role-play and humor used by the midwives providing the inspirational lectures have previously been shown to facilitate laughter among pregnant women and their partners. To a further extent, this laughter increases the parents' perceived abilities to absorb and understand the provided information (3, 4). Previously, humor has been associated with good health, possibly related to the release of endogenous hormones (endorphins, etc.) when laughter erupts (27). Whether or not healthcare professionals use humor may be related to both social and external circumstances, as well as to personality. Unwillingness to use humor within clinical practice could be caused by assumptions that humor is unprofessional (28). The midwives within the present study were aware of the importance of balancing humor with seriousness, to retain professionalism. Subsequently, midwives providing parental classes should present information in ways that help pregnant women and their partners to relax. This is because a feeling of being relaxed may facilitate the parents' abilities to understand information. For this reason, humor and practical illustrations could apparently be useful approaches. Nevertheless, it is valuable to bear in mind that the use of humor may not always be appropriate within care situations. Therefore, the use of humor within different international and cultural aspects may gain from further exploration.

However, expectant parents' views of childbirth differ. While some experience childbirth as a meaningful transformatory process, others experience it as a necessary part of life. The current findings illustrate how the midwives introduced childbirth as a normal life event for the expectant parents. Such an approach to childbirth could be likened to Bryar and Sinclair's (29) first model, that is: seeing pregnancy as a normal life event and as a period of growth for women, in contrast to the second model: seeing pregnancy as an illness and encouraging women to view themselves as patients (29). Pregnant women who strive to achieve normality are not strengthened by a focus on risk during childbirth (30). In addition, expectant parents need support to manage stressors that come with pregnancy. The parents need to identify both their internal and external resources required to manage those stressors. Therefore, a focus on risk during childbirth may hamper the expectant parents' abilities to manage the changes that come with childbirth. Approaching childbirth as a normal life event could be associated with salutogenesis, which aims to improve the parents' well-being or healthiness (31). Previously, Downe (32) has emphasized the need of midwives to use a more specific salutogenic approach. Furthermore, Bauer et al. (33) have proposed future salutogenic interventions to focus on strengthening individuals' resources, promoting their comprehensible, manageable, and meaningful life experiences as well as positive health outcomes. In relation to midwifery, such a salutogenic approach or intervention could be understood as midwives supporting the parents in experiencing childbirth as a comprehensible, manageable, and meaningful life experience. This is in line with the current findings. Therefore, midwives who possess valuable knowledge about how to preserve childbearing as a normal life event, should have a central role in supporting and maintaining childbearing as a period of growth for the parents. This is because midwives who are providing professional support, such as the inspirational lecture, with the intent to strengthen parents, should be seen as providing a vital care intervention aiming at promoting mental health and sustainability among parents.

Using a qualitative inductive design and content analysis (15) for the present study was deemed to be appropriate. Conducting telephone interviews was considered to be appropriate as well, since being interviewed via telephone was deemed to be less time consuming and therefore more comfortable by the interviewees (34). However, interviewing via telephone may be challenging for both the interviewer and the research process. This is because there is no way for the interviewer to analyze body language or facial expressions among the interviewees (35). However, the interviewer had a broad experience from conducting interviews via telephone and had the ability to be attentive to oral expressions. In case of obscurity, the interviewer asked about the specific participant's experiences from being interviewed. Overall, the authors had a consensus that the interviews were rigorous and met the aim of the present study. All the researchers with their different professional backgrounds participated in the analysis process and the findings have been illustrated with quotations. Furthermore, the final analyses were discussed with other researchers possessing a variety of experiences in working with professional support for expectant parents. This contributed to the trustworthiness of the study (36). The participants were a limited number of midwives using a specific method in a limited geographic region in Sweden, which has to be taken into consideration. However, since all eight midwives who provided the Inspirational Lecture as a parental class for the time of the study were included, it could be considered to be a study strength since the participants were representative and had knowledge about the studied phenomena (37). The way midwives provide professional support and parental classes for expectant parents vary, however, not only nationally within Sweden, but also internationally. Therefore, the results of the current study may not be directly transferrable to other settings. Nevertheless, qualitative research does not intend to generalize the results into other settings (38), instead the intent with qualitative research is to gain further knowledge about the phenomena studied within the contextual circumstances for the research participants. In addition, the results from the current study increase knowledge about midwives' experiences from providing parental classes for expectant parents. Subsequently, the results can assist midwives (or other health care professionals) and serve as a reference for providing parental classes for expectant parents.

However, since the inspirational lecture is a large-group parental class, it may be difficult to meet all the support needs of expectant parents in their preparation for childbirth. Previously, Gilmer et al. (39) have claimed that it would be impossible for a single and centrally regulated program to be flexible enough to actually meet the support needs of all expectant parents. In addition, the inspirational lecture has previously been shown to be valuable for parents when provided as a care intervention for expectant parents in combination with ordinary parental classes (8). Notable is, however, that the content, strategies and philosophy outlined in the findings of the current study may be commonly used for parental classes around the world. Also, one disadvantage of the format of the inspirational lecture is having a class of 80–120 parents (40–60 couples) which could make interaction and active participation challenging. A Danish randomized controlled trial found that small parental classes may increase childbirth self-efficacy (40), which could be due to the opportunities for the parents to interact with each other (3, 4, 41) because parents need to learn from other parents (42). Therefore, the inspirational lecture, as a support for expectant parents, should not exclude other types of professional support for expectant parents, instead, the inspirational lecture should be offered in combination with other types of professional support.

## Conclusion and Clinical Implications

The midwives' overall experiences about providing the inspirational lecture as a parental class entailed that they wanted to put childbirth into a comprehensive and manageable context for expectant parents. To achieve this, it was essential to make childbirth understandable, to promote confidence in the childbirth process and to facilitate the expectant parental couple's cooperation during childbirth. Further, the midwives strove to support the expectant parents in their understanding of how the parents themselves can become a “birthing team” and engaged participants in their upcoming birth. This brings valuable knowledge into the field, since it demonstrates how midwives strive to make expectant parents understand how the parents themselves can be engaged participants in their own birth. It could be understood as facilitating the integrative power that is the couples' ability to gather their joint power. Subsequently, current results revealed how midwives use different approaches to increase the parents' understanding. Such knowledge is of international relevance within clinical practice, since parental classes are offered by midwives globally. However, since the inspirational lecture is a large-group parental class, it may be difficult to meet all the support needs of expectant parents. Therefore, the inspirational lecture should be offered in combination with other types of professional support.

## Data Availability Statement

The datasets generated for this article are not readily available because we do not have permission to share data. Requests to access the datasets should be directed to caroline.backstrom@his.se.

## Ethics Statement

Ethical review and approval was not required for the study on human participants in accordance with the local legislation and institutional requirements. The patients/participants provided their written informed consent to participate in this study.

## Author Contributions

CB and TS contributed to the conceptualization, data curation, formal analysis, funding acquisition, investigation, methodology, project administration, resources, software, validation, visualization, and writing the article, which is an original draft. ST and LM contributed to the conceptualization, formal analysis, funding acquisition, investigation, methodology, project administration, resources, software, validation, visualization, and writing the article, which is an original draft. MG contributed to the conceptualization, data collection, formal analysis, funding acquisition, investigation, methodology, project administration, resources, software, validation, visualization, and writing the article, which is an original draft. All authors contributed to the article and approved the submitted version.

## Conflict of Interest

The authors declare that the research was conducted in the absence of any commercial or financial relationships that could be construed as a potential conflict of interest.
